# Stochastic Differential Equation Model for Cerebellar Granule Cell Excitability

**DOI:** 10.1371/journal.pcbi.1000004

**Published:** 2008-02-29

**Authors:** Antti Saarinen, Marja-Leena Linne, Olli Yli-Harja

**Affiliations:** Institute of Signal Processing, Tampere University of Technology, Tampere, Finland; University College London, United Kingdom

## Abstract

Neurons in the brain express intrinsic dynamic behavior which is known to be stochastic in nature. A crucial question in building models of neuronal excitability is how to be able to mimic the dynamic behavior of the biological counterpart accurately and how to perform simulations in the fastest possible way. The well-established Hodgkin-Huxley formalism has formed to a large extent the basis for building biophysically and anatomically detailed models of neurons. However, the deterministic Hodgkin-Huxley formalism does not take into account the stochastic behavior of voltage-dependent ion channels. Ion channel stochasticity is shown to be important in adjusting the transmembrane voltage dynamics at or close to the threshold of action potential firing, at the very least in small neurons. In order to achieve a better understanding of the dynamic behavior of a neuron, a new modeling and simulation approach based on stochastic differential equations and Brownian motion is developed. The basis of the work is a deterministic one-compartmental multi-conductance model of the cerebellar granule cell. This model includes six different types of voltage-dependent conductances described by Hodgkin-Huxley formalism and simple calcium dynamics. A new model for the granule cell is developed by incorporating stochasticity inherently present in the ion channel function into the gating variables of conductances. With the new stochastic model, the irregular electrophysiological activity of an in vitro granule cell is reproduced accurately, with the same parameter values for which the membrane potential of the original deterministic model exhibits regular behavior. The irregular electrophysiological activity includes experimentally observed random subthreshold oscillations, occasional spontaneous spikes, and clusters of action potentials. As a conclusion, the new stochastic differential equation model of the cerebellar granule cell excitability is found to expand the range of dynamics in comparison to the original deterministic model. Inclusion of stochastic elements in the operation of voltage-dependent conductances should thus be emphasized more in modeling the dynamic behavior of small neurons. Furthermore, the presented approach is valuable in providing faster computation times compared to the Markov chain type of modeling approaches and more sophisticated theoretical analysis tools compared to previously presented stochastic modeling approaches.

## Author Summary

Computational modeling is of importance in striving to understand the complex dynamic behavior of a neuron. In neuronal modeling, the function of the neuron's components, including the cell membrane and voltage-dependent ion channels, is typically described using deterministic ordinary differential equations that always provide the same model output when repeating computer simulations with fixed model parameter values. It is well known, however, that the behavior of neurons and voltage-dependent ion channels is stochastic in nature. A stochastic modeling approach based on probabilistically describing the transition rates of ion channels has therefore gained interest due to its ability to produce more accurate results than the deterministic approaches. These Markov chain type of models are, however, relatively time-consuming to simulate. Thus it is important to develop new modeling and simulation approaches that take into account the stochasticity inherently present in the function of ion channels. In this study, we seek new stochastic methods for modeling the dynamic behavior of neurons. We apply stochastic differential equations (SDEs) and Brownian motion that are also commonly used in the air space industry and in economics. An SDE is a differential equation in which one or more of the terms of the mathematical equation are stochastic processes. Computer simulations show that the irregular firing behavior of a small neuron, in our case the cerebellar granule cell, is reproduced more accurately in comparison to previous deterministic models. Furthermore, the computation is performed in a relatively fast manner compared to previous stochastic approaches. Additionally, the SDE method provides more sophisticated mathematical analysis tools compared to other, similar kinds of stochastic approaches. In the future, the new SDE model of the cerebellar granule cell can be used in studying the emergent behavior of cerebellar neural network circuitry.

## Introduction

Neurons express intrinsic bioelectrical activity which is known to be stochastic in nature. In order to understand this complex dynamic behavior, computational modeling is inevitable. But, how to develop models that are capable of mimicking the intrinsic dynamic behavior of the biological counterpart accurately? On the other hand, how can detailed models, possibly also incorporating some sort of stochasticity, be simulated in a reasonable time? These questions are crucial in creating computer models of neurons with better predictive capabilities.

It is well known that many components of a neuron and its membrane, including voltage-dependent ion channels, are essential for the dynamic behavior (see, e.g., [1]). Stochasticity may as well play an interesting role in the dynamic behavior of neurons [2],[3],[4],[5]. Recent studies have indicated that the primary source of stochasticity, or noise, in vivo is the synaptic input activity (see, e.g., [2],[6]). However, there are other noise sources as well (for a review, see, e.g., [7]), including extrasynaptic inputs and ion channel stochasticity, that can have significant implications on the dynamic behavior of neurons.

Several stochastic approaches have previously been developed for modeling the bioelectrical activity of neurons and excitable tissue. Monte Carlo simulations using discrete Markov chain type of models have been performed to understand the role of randomly opening ion channels (so called microscopic approach; [7],[8],[9],[10],[11],[12],[13],[14],[15],[16],[17],[18]). On the other hand, the so called “ODE plus white noise” approach (i.e., ordinary differential equation with additive white noise) and the Langevin equations have been exploited. In these approaches, noise has been incorporated into synaptic, conductance, or voltage equations of the deterministic models (so called macroscopic level; [2],[5],[7],[19],[20],[21] for synaptic, [16],[22],[23],[24],[25] for conductance, and [21],[26],[27] for voltage incorporation of noise). Regardless of the approach, all previous studies have emphasized the importance of stochasticity on firing (see [28]). Most of the previous studies have used simple deterministic model systems, including the Fitzhugh-Nagumo neuron model [27], the Morris-Lecar model, the Hindmarsh-Rose model [29], leaky integrate-and-fire model [5],[26],[30],[31], cable model [19], and the two-conductance Hodgkin-Huxley (H-H) model [7],[8],[10],[13],[14],[15],[16],[17],[20],[22],[23],[24],[25], as example systems to study the effects of stochasticity. Only a few previous studies [2],[9],[11],[12],[21],[32] have used more realistic deterministic models than the two-conductance H-H model.

Recent theoretical work has provided evidence that more emphasis should be put on ion channel stochasticity and its role in intrinsic dynamic behavior of neurons [8],[9],[10],[11],[12]. Ion channel stochasticity is due to the thermal interaction of molecules constituting an ion channel and it can be observed as random opening and closing (gating) of an ion channel at an experimentally fixed membrane potential. This probabilistic gating of an ion channel can be considered as “ion channel noise” or “ion channel stochasticity”. Several experimental studies have shown that the opening of a single ion channel can trigger action potentials in small excitable cells that have a high input resistance. These cells include small cultured bovine chromaffin cells [33], acutely isolated mouse [34] and rat [35] olfactory receptor neurons, small cultured hippocampal neurons [36], and small cultured cerebellar granule cells [37]. The total membrane current of a small neuron is influenced by ion channel stochasticity. This can change the transmembrane voltage dynamics at or close to the threshold of firing and affect action potential initiation and subthreshold membrane potential oscillations. Subthreshold oscillations may be important in determining the reliability and accuracy of action potential timing, as well as in coincidence detection and multiplication of inputs [10].

The well-established H-H formalism has formed, to a great extent, the basis for building biophysically and anatomically detailed models of neurons. Subsequently, the roles of conductances (and, ion channels) have been addressed using these models. It should be noted, however, that the deterministic H-H formalism does not take into account the fact that the behavior of ion channels underlying the whole-cell ionic currents is stochastic in nature. In other words, the ion channel stochasticity has been ignored, as also pointed out by White et al. [28] and Carelli et al. [12]. Instead, ionic conductances have been modeled as continuous, deterministic processes. In an effort to achieve a better understanding of the complex intrinsic dynamics of a single neuron, a new approach based on stochastic differential equations (SDEs) and Brownian motion is developed here. An SDE is a differential equation in which one or more of the terms are stochastic processes, thus resulting in a solution which is itself a stochastic process. The small, electrotonically compact cerebellar granule cell is used as an example to verify broader applicability of the SDE approach for modeling. For biophysical plausibility, the stochasticity is incorporated into the gating variables of all conductances in the compartmental H-H type of model for the cerebellar granule cell. Preliminary results of the work have been presented in [38].

## Materials and Methods

### Test Case

In this study, we use cerebellar granule cell as a test case and examine how the behavior of a small-size neuron is altered when stochasticity is introduced into the deterministic H-H type of model. In short, granule cells are glutamatergic excitatory neurons which translate the mossy fiber input into parallel fiber input to Purkinje cells [39],[40]. Granule cells are the smallest and the most numerous neuron type in the mammalian brain and have a simple morphology with an average of four short dendrites [39],[40], each receiving a single mossy fiber input. Previous experimental and modeling studies have shown that the granule cell has an electrotonically compact structure [41],[42]. This cell can thus be represented using only one compartment. Moreover, the basic single-neuron firing properties and the electroresponsiveness to various types of inputs, including intrasomatic pulses of currents and synaptic currents, have been extensively studied in vitro [43],[44],[45],[46] and in vivo [47] using the patch-clamp technique [48].

### Deterministic Model

Several deterministic models have been presented for the cerebellar granule cell during the past few years [42],[46],[49],[50]. When studying the behavior of these deterministic models (see also [51]), it has become clear that, with the given parameter values, the deterministic single-cell models are not capable of reproducing the experimentally observed irregular behavior in vitro in response to depolarizing current pulses. For example, the irregularity in interspike intervals during firing, or the subthreshold membrane oscillations observed in vitro in response to current pulses ([46], see also in vivo [47]), cannot be reproduced with the existing deterministic models in a straightforward manner.

In this study, we select to use the deterministic model of [50],[51] as the basis of our new stochastic model. The deterministic model is a parallel conductance, one-compartmental model previously developed for a cultured cerebellar granule cell. The model includes six different voltage-, time- and calcium-dependent ionic currents (Na_F_, K_Dr_, K_A_, K_ir_, Ca_HVA_, and BK_Ca_; Na_F_ stands for the fast inactivating sodium channel, K_Dr_ for the delayed rectifier potassium channel, K_A_ for the transient A-type potassium channel, K_ir_ for the inward rectifier potassium channel, Ca_HVA_ for the high-voltage-activated calcium channel, and BK_Ca_ for the large-conductance calcium- and voltage-activated potassium channel) and simple calcium dynamics to describe the changes in the membrane potential. The model is based on the theory of equivalent electrical circuits, as is conventionally done in neuronal compartmental modeling. The change in membrane potential, *V_m_*(*t*), is described using the ordinary differential equation
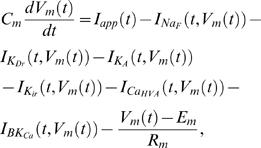
where *I*_app_(*t*) is the applied current (for the description of parameters, see Table 1). The behavior of ionic currents 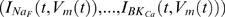 is described using algebraic equations according to the H-H formalism [52]. For example, for the Na_F_ channels, we have
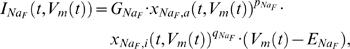
(2) is the maximal conductance of the Na_F_ channels, 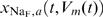 and 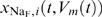 are the time- and voltage-dependent gating variables for the activation and inactivation processes of the Na_F_ channels, respectively. Furthermore, constants 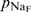 and 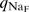 are the exponentials for the corresponding activation and inactivation processes, and 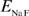 the equilibrium potential for Na^+^. The processing of calcium ions is assumed to take place in small volume close to cell membrane and is linked to BK_Ca_ channel function. The change in intracellular calcium concentration, [Ca^2+^], is described by
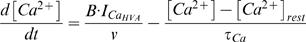
(3)where 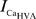 is the current of the Ca_HVA_ channels and *v* is the volume in which calcium ions are processed. For the parameters *B*, [Ca^2+^]_rest_, and τ*_Ca_*, see Table 1.

**Table 1:** Parameter values used in both stochastic and deterministic simulations.

Constant	Value	Description
*R_m_*	0.57 Ωm^2^	membrane resistance
*C_m_*	0.03 F/m^2^	membrane capacitance
*E_m_*	−0.025 V	equilibrium membrane potential
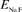	+0.07 V	equilibrium potential for Na^+^
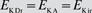	−0.075 V	equilibrium potential for K^+^
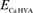	+0.14 V	equilibrium potential for Ca^2+^
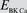	−0.085 V	equilibrium potential for *BK_Ca_*
*B*	5.2⋅10^−6^ mol/C	constant for Ca^2+^ transfer into the cell
[Ca^2+^]_rest_	100⋅10^−6^ mol/m^3^	[Ca^2+^] at rest
τ*_Ca_*	1⋅10^−3^ s	time constant for the decay of
		intracellular free calcium
dcell	6⋅10^−6^ m	diameter of the granule cell
dshell	1⋅10^−7^ m	diameter of the shell defining the volume
		in which calcium ions are processed
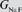	400 S/m^2^	maximal conductance for Na_F_
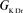	120 S/m^2^	maximal conductance for K_Dr_
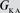	10 S/m^2^	maximal conductance for K_A_
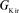	28 S/m^2^	maximal conductance for K_ir_
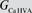	4.6 S/m^2^	maximal conductance for Ca_HVA_
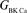	30 S/m^2^	maximal conductance for BK_Ca_
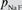	3	exponential for Na_F_ activation
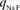	1	exponential for Na_F_ inactivation
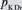	4	exponential for K_Dr_ activation
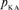	3	exponential for K_A_ activation
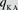	1	exponential for K_A_ inactivation
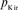	1	exponential for K_ir_ activation
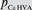	2	exponential for Ca_HVA_ activation
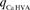	1	exponential for Ca_HVA_ inactivation
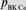	1	exponential for BK_Ca_ activation

See the sections Deterministic Model and Complete Stochastic Model for more details on ion channel types and the description of the complete mathematical model.

**Figure 1:**
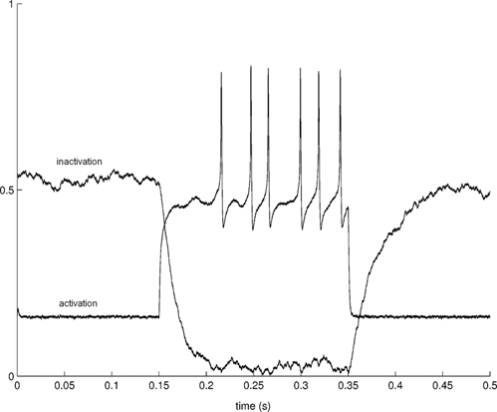
Gating variables for K_A_ channel activation and inactivation processes. The stochastic model is simulated for 0.5 seconds and depolarized from 0.15 seconds to 0.35 seconds. The value of the parameter σ is set to 0.15 and all other parameters are fixed as explained in the text.

**Figure 2:**
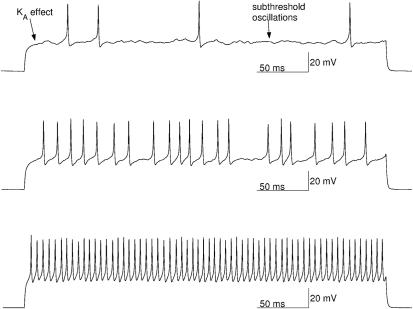
The behavior of the stochastic granule cell model in response to three different depolarizing current pulses. In each trace, firing is simulated for 0.4 seconds with a time step of 10^−5^ seconds. In the upper panel the depolarizing current is below firing threshold (*I_app_ = *11 pA) and in the middle panel just above firing threshold (*I_app_* = 12 pA). In the lower panel, the depolarizing current is considerably larger (*I_app_* = 29 pA). The upper trace illustrates the spontaneous firing occasionally present when a depolarizing current below firing threshold is given. The middle trace shows the irregularity in firing with small depolarizing current pulses. The two uppermost traces also contain random subthreshold oscillations. With larger depolarizing current pulses, firing becomes more regular, as shown in the lower panel. Due to stochastic nature of the model, the interspike intervals and the height of action potentials also show slight irregularity. In each case σ = 0.5 and all other parameters are fixed as explained in the text.

The parameter values of the original deterministic model have been selected based on data taken from in vivo and in vitro experimental records (for references see [50],[51]) on cerebellar granule cells. The original deterministic model has been verified in detail against the electrophysiological data recorded from in vitro granule cells (cf. Figures 5.3, 5.4, 5.5, 5.6, and 5.7 in [51]; cf. Figures 1, 2, and 3 in [50]). A semi-automatic parameter estimation procedure to fit the model to in vitro current clamp data is presented in [50],[51]. See [50],[51] for more details of the construction and fine-tuning of the original deterministic model. It has been shown that the deterministic model reproduces the basic firing properties of an in vitro granule cell, such as the frequent firing, the correct frequency-current (*f-I*) curve with different depolarizing current pulses, and the realistic single action potential waveform in response to intrasomatic current pulses [50],[51]. The deterministic model has been previously simulated using GENESIS simulator software [53]. In summary, we employ **(i)** a realistic one-compartmental H-H type of model, **(ii)** six voltage-dependent ionic conductances, **(iii)** simplified calcium dynamics, and **(iv)** stochasticity in the gating variables of ionic conductances. Item **(iv)** is further described in the next section.

**Figure 3:**
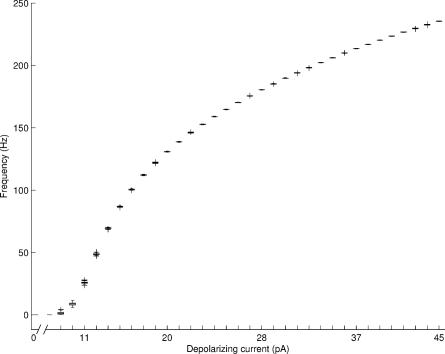
Frequency-current (*f-I*) curve of the stochastic granule cell model presented as a box and whisker plot. Depolarizing current pulses from 0 pA to 45 pA are used. For each value of depolarizing current we simulated 50 realizations, each 50 seconds long. Median, upper and lower quartiles, and the maximal and minimal firing frequencies are given for each depolarizing current pulse; outliers are marked with+symbol. Spontaneous activity is observed at low firing frequencies with depolarizing currents below 11 pA which is the firing threshold of the model. The *f-I* curve of the stochastic model is linear up to a frequency of 125 Hz, after which it shows saturation. For every realization σ = 0.5.

## Results

### Development of the Stochastic Model

The random nature of synaptic activity, including the probabilistic release of neurotransmitters from synaptic vesicles, is one of the main sources of noise causing variability of firing. When modeling neuronal dynamics, stochasticity has thus been typically incorporated in the model input (see, e.g., [2],[5],[19],[26]), not integrated into the model. The role of synaptic processes, however, is not covered in the present study. Instead, we concentrate on studying the random behavior of voltage-gated ion channels in shaping the input-output relations and the intrinsic dynamics of a neuron.

There are alternative ways of introducing stochasticity in the behavior of the voltage-gated ion channels. In this work, we approximate the randomness in the operation of voltage-dependent ion channels as Brownian motion, i.e., as a Gaussian process with independent increments. Therefore, we convert the complete deterministic model for the cerebellar granule cell into a stochastic model. We describe the activation and inactivation of the six different ionic conductances using stochastic differential equations of the form
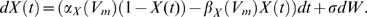
(4)


Here, the original deterministic equation [52] is modified by adding the stochastic component σd*W*. In the Equation 4, *X*(*t*) denotes the gating variable for the ion channel type in question, α*_X_* and β*_X_* the rate functions of activation or inactivation processes, and *W* Brownian motion. Brownian motion thus models the effects of random openings and closings of ion channels known to contribute to the very delicate subthreshold membrane dynamics in neurons. In our stochastic model, the parameter σ allows us to take into account the intensity of random fluctuations. Equation 4 is a short-hand notation of the corresponding integral equation of the form
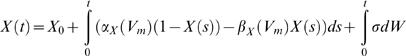
(5)where the last stochastic integral is interpreted as Itô-integral with respect to Brownian motion. To our knowledge this mathematical approach has not been presented before for realistic compartmental models of neurons, other than the cerebellar granule cell [38].

Using the common alternative notation, Equation 4 could also be given in the form
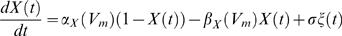
(6)which includes the theoretically problematic variable, the “white noise process” ξ(*t*). In this paper, however, we interpret Equation 6 as a short-hand notation for Equation 5 and give an example how Equation 5 is used in the previous stochastic expansions of the original H-H model. For example, Fox [22] uses, in contrast to our model (Equation 4), a specific form of autocorrelation function to characterize the dynamics of ξ(*t*). This autocorrelation function has the form
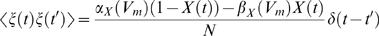
(7)where *N* is the number of specific ion channels on a given area. This form of autocorrelation function implies that ξ(*t*) is no longer white noise, and the solution to corresponding equation (Equation 5) can no longer be interpreted as an Itô-integral with respect to Brownian motion.

Specific types of autocorrelation functions have been used to avoid values for the gating variables which are not in the interval [0,1]. Autocorrelation function has been constructed so that it decreases the variance of the stochastic component when the value of a gating variable approaches 0 or 1. Although this approach decreases the probability of obtaining values outside the desired range, there is still a possibility that in a given point the realization of the stochastic component results in a value of the gating variable not in the interval [0,1].

It is possible to completely avoid values for the gating variables which are not in the interval [0,1]. The use of reflecting boundaries (i.e., the values under 0 or over 1 are reflected back to interval [0,1]) prevents the undesired values, but results in a model which does not correspond to the original stochastic integral equation (Equation 5).

In our model, we use a constant parameter σ and increments of Brownian motion, which ensures that the produced realizations are truly solutions of the corresponding integral equation. Another reason for selecting a constant parameter σ to our model is that, in the future, we are able to estimate its value using maximum likelihood estimation methods. This kind of estimation would be more difficult for a time-dependent parameter σ.

We have to be concerned about the undesired values of the gating variables, because the stochastic component in our model has now constant variance. This would result in problems when the values of the gating variables are close to 0 or 1. However, we are able to almost completely avoid undesired values for the gating variables by properly controlling the value of parameter σ. During depolarization only the gating variable for the K_A_ channel inactivation approaches zero and large negative values of the stochastic component would result in negative values of the gating variable. Hence, we have to use small values of parameter σ or use a separate parameter describing the stochastic fluctuations in the K_A_ channel inactivation process. For this paper, we choose the former approach and use the same, small value of parameter σ for all activation and inactivation processes. When the model is not depolarized, some of the gating variables are fluctuating relatively close to zero or one. This also limits our choice of proper value for the parameter σ.

In Figure 1, we present the gating variables for K_A_ activation and inactivation process. From Figure 1 it can be seen that the model behavior is stable when the model is not depolarized, and during depolarization a properly selected value for the parameter σ ensures that the values for the gating variable are in the interval [0,1].

### Complete Stochastic Model

The complete stochastic model used in this work is described with Equation 8. We use our independently developed simulation software in the MATLAB programming environment to make the calculations. The random numbers required in the simulations are generated with MATLAB's random number generators. The following equations are used to calculate the change in membrane potential, *V_m_*, in intracellular calcium concentration, [Ca^2+^], and in the gating variables for activation and inactivation processes at each time point
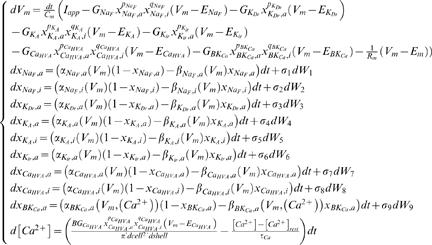
(8)The parameters for the equations are given in Table 1 and the rate functions for the gating variables in Table 2. The selection of parameter values, including those in the rate functions, is explained in the Deterministic Model section and in [50],[51].

**Table 2:** Forward and backward rate functions for different ion channel types in the stochastic model (see Equation 8).

Channel	Process	Forward rate function	Backward rate function
Na_F_	activation	α_NaF,a_(V_m_) = 3•10^3^•*e*((V_m_−0.01)+39•10^−3^)•0.081•10^3^	β_NaF,a_(V_m_) = 3•10^3^•*e*((V_m_−0.01)+39•10^−3^)•−0.066•10^3^
Na_F_	inactivation	α_NaF,i_(V_m_) = 0.24•10^3^•*e*((V_m_−0.01)+50•10^−3^)•−0.089•10^3^	β_NaF,i_(V_m_) = 0.24•10^3^•*e*((V_m_−0.01)+50•10^−3^)•0.089•10^3^
K_Dr_	activation	α_KDr,a_(V_m_) = 0.34•10^3^•*e*((V_m_−0.01)+38•10^−3^)•0.073•10^3^	β_KDr,a_(V_m_) = 0.34•10^3^•*e*((V_m_−0.01)+38•10^−3^)•−0.018•10^3^
K_A_	activation	α_KA,a_(V_m_) = 2.2•10^3^•*e*((V_m_−0.01)+46.7•10^−3^)•0.04•10^3^	β_KA,a_(V_m_) = 2.2•10^3^•*e*((V_m_−0.01)+46.7•10^−3^)•−0.01•10^3^
K_A_	inactivation	α_KA,i_(V_m_) = 0.016•10^3^•*e*((V_m_−0.01)+78.8•10^−3^)•−0.075•10^3^	β_KA,i_(V_m_) = 0.016•10^3^•*e*((V_m_−0.01)+78.8•10^−3^)•0.055•10^3^
K_ir_	activation	α_Kir,a_(V_m_) = 0.133•10^3^•*e*((V_m_−0.01)+83.94•10^−3^)•−0.0411•10^3^	β_Kir,a_(V_m_) = 0.17•10^3^•*e*((V_m_−0.01)+83.94•10^−3^)•0.028•10^3^
Ca_HVA_	activation	α_CaHVA,a_(V_m_) = 0.049•10^3^•*e*((V_m_−0.01)+29.06•10^−3^)•0.063•10^3^	β_CaHVA,a_(V_m_) = 0.082•10^3^•*e*((V_m_−0.01)+18.66•10^−3^)•−0.039•10^3^
Ca_HVA_	inactivation	α_CaHVA,i_(V_m_) = 0.0013•10^3^•*e*((V_m_−0.01)+48•10^−3^)•−0.055•10^3^	β_CaHVA,i_(V_m_) = 0.0013•10^3^•*e*((V_m_−0.01)+48•10^−3^)•0.012•10^3^
BK_Ca_	activation	α_BKCa,a_(V_m_,[Ca^2+^]) = (2.5•10^3^)/(1+1.5•10^−3^•*e*(−0.085•10^3^•(V_m_−0.01))/[Ca^2+^])	β_BKCa,a_(V_m_,[Ca^2+^]) = (1.5•10^3^)/(1+[Ca^2+^]/(150•10^−6^ •*e*(−0.077•10^3^•(V_m_−0.01))))

In the model, *W_i_* = {*W_i_*(*t*),*t*≥0} is Brownian motion (sometimes called the standard Wiener process to distinguish between the mathematical and physical processes), that is a Gaussian process with independent increments. This means that all finite-dimensional distributions of Brownian motion are Gaussian, *W_i_*(0) = 0 almost surely, *E*(*W_i_*(*t*)) = 0 for all *t*≥0, and Var(*W_i_*(*t*)−*W_i_*(*s*)) = *t*−*s* for all *t*≥*s*≥0. In addition, *dW_i_* stands for the infinitesimal increment of Brownian motion. In the simulations, the increments of Brownian motion are created by sampling a zero-mean, unit-variance normal distribution after which the sample is scaled using the time-step of the simulation. Details on discretizing Brownian motion and stochastic differential equations can be found in [54],[55].

In stochastic simulation, we use the same parameter values as for the original deterministic model (Tables 1 and 2) to elucidate the effects of addition of parameters σ*_i_* on the dynamic behavior of the granule cell. For the parameters σ*_i_*, we assume that σ*_i_* = σ for *i* = 1,…,9. We use the Euler-Maruyama method [55] for simulating different realizations of the system. All simulations are carried out using the time-step Δ*t* = 10^−5^ s.

Using this stochastic H-H type of model (see Equation 8), we are able to simulate, by intrinsic properties of the model, the following dynamic behavior **(i)**–**(xii)**. The properties **(i)** through **(iv)** can be reproduced with both the deterministic and the stochastic model, while the properties **(v)** through **(xii)** only with the stochastic model. The stochastic expansion of the deterministic model retains all the properties of the deterministic model.

### Electroresponsiveness Obtained by Both Models

In the simulations, we observe **(i)** normal firing (Figure 2) that produces **(ii)** linear *f-I* curve with small depolarizing currents (Figure 3). The linearity of the *f-I* curve is an important requirement for a model of the cerebellar granule cell when small depolarizing current pulses are used [45],[46],[47],[51],[50]. Both the deterministic and stochastic models start firing when a small depolarizing current pulse of 11 pA is applied to the neuron soma, the value which is close to the experimentally observed threshold of firing found in vitro (cf. Figure 1B in [45]), see also in vivo (cf. Figure 1G in [47]). The *f-I* curves of the models are linear up to a frequency of 125 Hz, with no dampening of action potential amplitudes. With relatively strong depolarizing current pulses the models are still firing but show saturation of the *f-I* curves, due to high firing frequency of a small neuron.

The highest firing rate the models can attain is approximately 300 Hz. Firing frequencies of up to 250 Hz have been observed with little or no adaptation of firing in response to strong depolarizing current pulses in in vivo granule cells [47]. Furthermore, both models are capable of reproducing **(iii)** the K_A_ effect (Figure 2), which is a delay in the firing caused by the K_A_ current shown to exist in in vitro granule cells [44],[45], see also in vivo [47]. Also **(iv)** fast afterhyperpolarizations (fAHP) are reproduced realistically with both models.

### Electroresponsiveness Obtained by the Stochastic Model Only

Experimental findings have indicated that irregularities in the firing of cerebellar granule cells are at least partly driven by intrinsic mechanisms, not exclusively by synaptic mechanisms. Irregularity in firing, as well as random subthreshold membrane oscillations, have been measured in the presence of 10 µM bicuculline blocking GABA-ergic inhibition [46]. Moreover, spontaneous excitatory postsynaptic potentials (EPSPs) have rarely been detected in these experiments [46]. Similarly, irregularity in firing has been measured after application of the glutamate receptor blockers (10 µM CNQX, 100 µM APV, and 50 µM 7-Cl-kyn) [46]. Also, subthreshold membrane oscillations have been found to be independent from synaptic activity [45].

**Figure 4:**
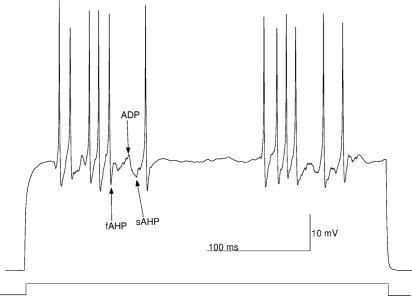
Exploring the intrinsic burst generation with the stochastic granule cell model. A small depolarizing current pulse (shown by a rectangular bar at the bottom of the figure) below firing threshold is injected into the cell soma. The bursts are evoked by random changes of σ between the values σ = 0.3 and σ = 1.1 (i.e., during a burst the value of parameter σ is increased to 1.1 otherwise it being 0.3). For illustrative purposes the trace with two bursts of action potentials is shown here (compare also with Figure 5). A fast afterhyperpolarization (fAHP), an afterdepolarization (ADP), and a slow afterhyperpolarization (sAHP) are indicated by arrows.

As an improvement to the deterministic granule cell model considered in this work [50],[51], and to the other previously presented deterministic models for cerebellar granule cells [42],[46],[49], we are now able to reproduce with fixed parameter values **(v)** irregularity in firing, including clusters of action potentials, **(vi)** random subthreshold membrane oscillations, and **(vii)** variability in heights of action potentials (Figure 2). These firing properties have been shown to be present in vitro (cf. Figures 2A and 2B in [45]; cf. Figures 1A and 1B in [46]), see also in vivo (cf. Figures 1C, 1D, and 1F in [47]). Furthermore, **(viii)** afterdepolarizations (ADP) and **(ix)** slow afterhyperpolarizations (sAHP) are reproduced realistically with small depolarizing current pulses (Figure 4; cf. Figure 1B in [46]).

**Figure 5:**
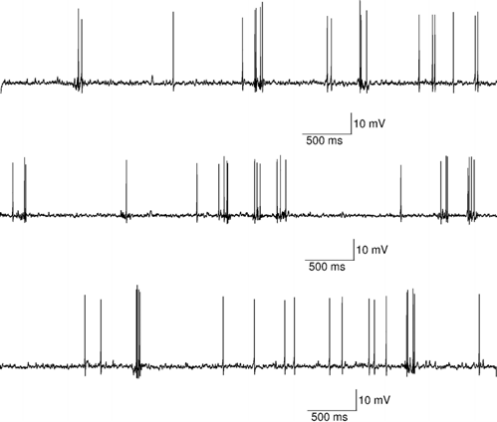
Dynamic behavior of the stochastic granule cell model simulated for a longer duration (15 seconds). A small depolarizing current below firing threshold is applied throughout the simulation, similarly as in Figure 4. Bursts and occasional spontaneous firing can be observed. Bursts are evoked by random changes of σ between the values σ = 0.3 and σ = 1.1 (during a burst the value of parameter σ is increased to 1.1 otherwise it being 0.3). This 15-second simulation also provides evidence that stable solutions are obtained when bursts are evoked.

Occasional **(x)** spontaneous firing can also be observed with current pulses smaller than 11 pA, due to the stochastic nature of the model (Figure 2 (upper panel) and Figure 5). Granule cells have been shown not to be spontaneously active in in vitro slice preparation [45]. However, in vitro granule cells in culture [37], as well as in vivo granule cells [39],[47], have been shown to be able to generate spontaneous activity when tonic inhibition of Golgi cells is reduced.

**Figure 6:**
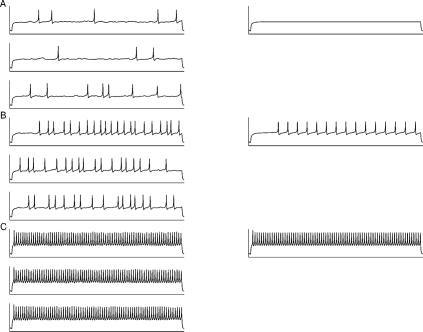
Comparison between the responses obtained by the deterministic and stochastic models. The length of each trace is 0.4 seconds. In A) the depolarizing current is below firing threshold (*I_app_* = 11 pA) and in B) just above firing threshold (*I_app_* = 12 pA). In C), the depolarizing current is considerably larger (*I_app_ = *29 pA). The deterministic model (right panels) does not reproduce the experimentally observed irregularity in firing. The responses simulated by the stochastic model (left panels), on the contrary, very closely resemble the experimentally obtained irregularities (for more details, see the Electroresponsiveness Obtained by the Stochastic Model Only section). For the stochastic traces σ = 0.5. For each value of the depolarizing current, *I_app_*, traces from three independent simulations of the stochastic model are shown to illustrate the variability of firing.

**Figure 7:**
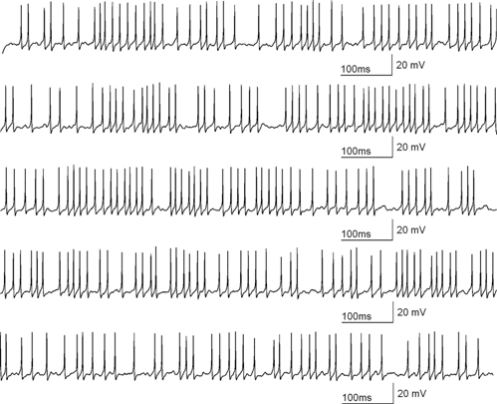
Example of stable, long-lasting, continuous simulation with irregular firing using the stochastic model. A depolarizing current pulse just above the firing threshold is given. A simulation of 5 seconds is shown to provide evidence that stable solutions are obtained with stochastic differential equations and Brownian motion. The simulation time of this trace with time-step of 10^−5^ seconds is ca. 15 seconds. For this simulation σ = 0.5.

A comparison between the responses obtained by the deterministic and stochastic model is shown in Figure 6. As can be seen from Figure 6, the deterministic model (right panels) does not reproduce the experimentally observed irregularity in firing. The responses simulated by the stochastic model of this study, on the other hand, very closely resemble the experimentally obtained responses. To show variability, traces from three independent simulations with the same initial conditions are shown. The stochastic model thus expands the dynamic range of one-compartmental multi-conductance model for the cerebellar granule cell in vitro. The term “dynamic range” used in this work does not refer only to the range of firing frequencies of the model, but to the whole range of different dynamic behaviors the model is capable of attaining. Furthermore, the use of SDE approach and the presence of Brownian motion does not lead to unstable results when simulating the stochastic granule cell model. As a demonstration of this two examples showing a longer, continuous simulation are plotted in Figure 5 and Figure 7.

### Analysis of Interspike Intervals for the Stochastic Model

**Figure 8:**
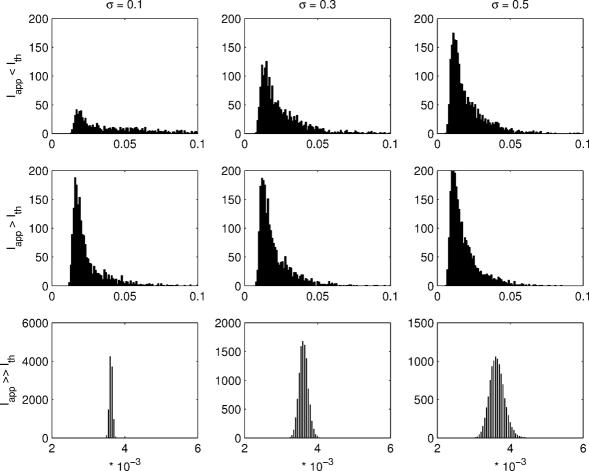
Histograms of interspike intervals. Firing is simulated for 50 seconds with each depolarizing current pulse, *I_app_*, and value of parameter σ. Three different values for depolarizing current and for the parameter σ are used. Three upper panels show firing with depolarizing current below the firing threshold (*I_app_* = 11 pA). Middle panels show firing with depolarizing current pulse just above the firing threshold (*I_app_* = 12 pA). Lover panels show firing with considerably larger depolarizing current pulses (*I_app_* = 29 pA). Note the different scales for the last row for illustrative purposes.

Variability in the firing caused by the parameter σ can be assessed by examining the histograms of interspike intervals with different values of depolarizing current pulses and different values of parameter σ (Figure 8). The histograms reveal that the value of parameter σ has a major effect on the firing with current pulses near the threshold of firing. With larger current pulses firing becomes more regular and the value of σ does not have as clear an effect. This can be observed from the histograms as a smaller deviation in the interspike intervals.

The existence of spontaneous firing can also be observed from Figure 8 (first row) where the applied current is below the threshold of firing. The increase in the value of parameter σ generates more and more spontaneous spikes which can be observed as an increase in the amount of small interspike intervals in Figure 8.

The coefficient of variation (CV) of the interspike intervals is often employed to quantify the regularity/irregularity of action potential firing. A completely regular firing has a CV of zero. In this work, the dependence of CV on different values of parameter σ and different depolarizing current pulses is studied. For the parameter values of σ = 0.1, 0.3, and 0.5, the results obtained for the mean, standard deviation (std), and CV are given in Table 3. Examination of the results shows variability in the mean firing rate when changing the value of parameter σ with depolarizing current pulses near the threshold of firing. Larger depolarizing current pulses cause the stochastic model to fire similarly as the deterministic model. With depolarizing current pulses above the threshold of firing (*I_app_* = 12 pA and *I_app_* = 29 pA; see Table 3), the increase in the value of parameter σ increases the irregularity of firing measured with the CV. However, with depolarizing current pulses below the threshold of firing (*I_app_* = 11 pA), the increase in the values of parameter σ enhances spontaneous activity, thus making the firing more regular. In other words, the increase in the value of parameter σ causes the membrane potential to pass the firing threshold more frequently thus decreasing the variability in the lengths of interspike intervals. This results in smaller values of CV when the value of parameter σ is increased. This can be seen from the CVs in Table 3.

**Table 3:** Quantitative analysis of the interspike intervals of the stochastic model.

Depolarizing current	σ	mean (s)	std (s)	CV
*I_app_* = 11 pA	0.1	0.0536	0.0461	0.8598
	0.3	0.0251	0.0155	0.6194
	0.5	0.0205	0.0124	0.6055
*I_app_* = 12 pA	0.1	0.0247	0.0130	0.5282
	0.3	0.0208	0.0118	0.5655
	0.5	0.0184	0.0106	0.5794
*I_app_* = 29 pA	0.1	0.0036	4.53⋅10^−5^	0.0125
	0.3	0.0036	1.24⋅10^−4^	0.0343
	0.5	0.0036	2.04⋅10^−4^	0.0562

Firing is simulated for 50 seconds with three different values of depolarizing current pulses, *I_app_*, and the parameter σ. The chosen levels for depolarizing current pulses are: **i)** *I_app_* = 11 pA (below firing threshold, *I_th_*), **ii)** *I_app_* = 12 pA (just above the firing threshold), and **iii)** *I_app_* = 29 pA (a considerably larger stimulus). From each trace the mean, standard deviation (std) and the coefficient of variation (CV) of the interspike intervals are calculated. Seconds are used as units for the mean and standard deviation; coefficient of variation is dimensionless. Same simulated data is used as in Figure 8.

### Exploring the Possibilities for Burst Generation and Variability in Spike Timing with the Stochastic Model

Bursts of action potentials have been recently recorded in in vivo granule cells in response to sensory stimuli using patch-clamp technique (cf. Figures 3B and 3F in [47]). We are interested if these bursts can be generated intrinsically in in vitro cells, specifically in the light of recent findings by D'Angelo et al. [45]. In their study on in vitro granule cells, D'Angelo et al. [45] have concluded that bursting in cerebellar granule cells persists after NMDA receptor block (100 µM APV+50 µM 7-Cl-Kyn is used), indicating that the NMDA currents are not involved. By incorporating time dependency in the parameter σ, we are able to simulate **(xi)** bursts of intrinsic origin (Figures 4 and 5). In this study, we induce random changes in the parameter σ between two specified values. These values enable us to take into account two intensity levels of random fluctuations to obtain bursts. In the future, these changes can be implemented in such a way that they are linked with the experimentally observed fluctuations of, for example, voltage-dependent ion channels or synaptic currents, depending on which source(s) the bursting behavior arises.

**Figure 9:**
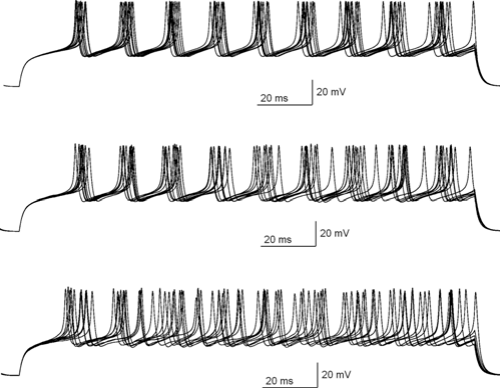
Exploring the variability in spike timing. Three sets of ten realizations of firing are simulated with the stochastic granule cell model using a depolarizing current pulse just above the firing threshold (*I_app_* = 12 pA). The length of each trace is 0.1 seconds. In the upper panel σ = 0.1, in the middle panel σ = 0.3, and in the lower panel σ = 0.5. The main variability does not arise only from the timing of the first action potential, but there is significant variability also after the first spike.

The **(xii)** variability in spike timing can be observed in repeated simulations with the same initial condition. As can be seen from Figure 9, the value of parameter σ affects spike timing. Figure 9 shows that the main variability does not arise only from the timing of the first action potential, but that there is significant variability also after the first spike.

Based on the simulation results presented in the last four sections, it can be concluded that our new stochastic model is capable of reproducing the details of the firing shown for granule cells in vitro [45],[46], see also in vivo [47].

### Computation Time

In addition to putting emphasis on choosing the correct noise model, there is a need to consider computational efficiency, especially with realistic neuron models. Using the same simulation environment, the computation time of the SDE model is only two times the computation time of the deterministic model. In other words, the simulations of the SDE model can be run in a time-scale of seconds with a standard desktop PC (in our simulations, 1.86 GHz processor with 2 GB of RAM). For example, simulating a five-second trace for Figure 7 (i.e., 500,000 time-points) using MATLAB (version 7.4.0.287 (R2007a)) programming environment takes ca. 15 seconds, in comparison to ca. 8 seconds of the deterministic model in the same simulation environment. Detailed benchmarking of different stochastic methods is demanding, being a topic of another study. It will require a careful implementation of methods using a suitable test case such as the H-H model of squid axon (see also Computation Time section in Discussion).

## Discussion

We have shown here that, by using stochastic differential equations and Brownian motion to incorporate ion channel stochasticity, it is possible to reproduce with high precision the intrinsic electrophysiological activity of a neuron. The method presented here has several advantages over deterministic and other stochastic approaches. First, the approach provides models of neurons with realistic irregular behavior better than the deterministic approaches commonly used in computational neuroscience. Second, it decreases the computation time in comparison to discrete stochastic approaches. Additionally, the method provides more sophisticated mathematical analysis tools compared to other, continuous stochastic approaches. In the following, we discuss these advantages as well as the limitations of the proposed method and point out some possible extensions for future work.

### Accurate Reproduction of Irregular Neuronal Activity

In general, there are a number of ways to improve deterministic compartmental models and to make them more accurate and realistic, as has also been pointed out by Carelli et al. [12]. One can include new conductances characterized for the neuron in question or introduce new dynamics for the existing conductances. Also calcium dynamics, among others, can be compartmentalized, and internal calcium stores can be added. We have strong confidence that it is equally important to consider alternative ways, such as the inclusion of stochasticity, to improve the compartmental models.

As there are experimental findings showing that irregular behavior observed in an in vitro granule cell may be driven by intrinsic mechanisms ([45],[46], see also the section Electroresponsiveness Obtained by the Stochastic Model Only), it is critical to consider ways to improve the deterministic model of the granule cell. With our new SDE model, irregularities in firing, inherent variability in electroresponsiveness and spike timing, as well as random subthreshold membrane oscillations, can be reproduced accurately. This is achieved by incorporating a stochastic component σd*W* in the deterministic equation for the gating variables and without changing any of the parameter values of the original deterministic model. In other words, the SDE model is able to reproduce the experimentally observed irregular behavior with the same parameter values for which the membrane potential of the original deterministic model exhibits regular behavior. Proper inclusion of stochastic elements in the operation of voltage-dependent ionic conductances should therefore be considered important, at the very least, for modeling the intrinsic electrophysiological activity of a small-size neuron.

Although several stochastic methods have been presented for describing the intrinsic activity of neurons (for a review, see, e.g., [28]), these methods have not been widely utilized in computational neuroscience, most probably due to computational reasons. At the microscopic level, a typical approach has been to use a chain of Markovian states with transition probabilities given directly by the H-H voltage-dependent transition rates (see, e.g., [8],[12],[18]). This kind of approach needs to be employed when the goal of the modeling study is to understand the biophysical mechanisms of ion channel gating. The SDE approach, on the other hand, can be used to describe the irregular behavior of a small neuron using the macroscopic measurements of ionic currents as such, thus avoiding the computationally demanding descriptions of single ion channel gating.

The computationally fast, yet accurate SDE model of the granule cell could be useful in studying the emergent behavior of cerebellar neural network circuitry. There are several interesting, experimentally observed phenomena that have to be addressed in the future, including the low-frequency oscillations observed in the cerebellar granule cell layer of awake, freely behaving rats [56] and anesthetized cats [39]. Furthermore, the tuning mechanisms controlling oscillations, resonant synchronization, and learning are of interest [47],[57],[58]. The SDE approach, in general, will help in simulating stochastic large-scale models in a relatively fast manner compared to many other stochastic approaches and in linking more tightly the molecular (see also [59]), cellular, network, and behavioral correlates of information processing in neural systems [60].

### Computation Time

In addition to accurate reproduction of experimental findings, it is important to consider the computation time required by a specific stochastic approach. In many cases, lack of computing resources has prevented the use of stochasticity in detailed compartmental modeling. Moreover, there are very few studies reporting actual computation times to benchmark existing stochastic methods and to guide the selection of suitable method. Carelli et al. [12] have made a conclusion that intensive computation is needed to study the stochastic Markov chain model of crustacean stomatogastric ganglion neuron and the simulation of long time-series can thus become infeasible. Faisal and Laughlin [18] have studied stochastic effects of action potential propagation in thin axons where ion channel gating has been described by discrete-state Markov processes, thus directly capturing the kinetics of ion channels from patch-clamp experiments. The calculation of stochastic effects, however, has been shown to require several months of computation time on a workstation cluster.

The computation time of our SDE model is, in contrast, only two times the computation time of the deterministic model. Therefore, the computation time is considerably decreased in comparison to discrete-state stochastic approaches in which the ion channels' transition rates are described as discrete-state Markov processes. The SDE method thus makes it possible to simulate long time series, similarly as in Figure 3, in a reasonable time.

### Theoretical Tools

One advantage of the SDE approach is that the approach provides more sophisticated theoretical tools for analysis of models in comparison to other previously presented continuous-state stochastic approaches (see, e.g., [54],[55]). For example, the computationally fast “ODE plus white noise” approach is limited to simulation purposes and does not provide as sophisticated mathematical tools as the SDE method. Examples of the theoretical tools for the SDE approach include Sequential Monte Carlo (SMC) simulation based maximum-likelihood (ML) estimation of the model parameters. SMC methods offer, in general, a set of methods which are very flexible, relatively easy to implement, parallelizable, and applicable universally.

SMC simulation based ML estimation is a Bayesian type of estimation technique which relies on transforming the probability distributions of the estimation problem into distributions which are easy to sample. This transformation allows us to use SMC approach when drawing samples from the desired posterior distributions. Based on these samples, a maximum-likelihood estimation technique is utilized for producing ML estimates for the selected model parameters. As an example, these parameters can include maximal conductances of ionic currents and the intensity of random fluctuations in the current-clamp data. This kind of fitting makes it possible to use irregular learning data in the estimation. Our ongoing work using the SDE version of the H-H model for a squid axon has shown that accurate ML estimates can be obtained for the selected model parameters based on irregular learning data [61]. Moreover, the approximation of the likelihood function allows us also to study the sensitivity of the model parameters and the effects of the changes in their values to the model behavior. The sharper the peak is in the likelihood, around the correct parameter value, the more sensitive the model behavior is with respect to value of that parameter.

### Challenges for Future Work

The SDE approach, inevitably, has certain challenges that need to be addressed in the future. First, the gating variables of the H-H type of models may have undesired values if no attention is paid to the selection of the value for the parameter σ. This problem may be corrected by implementing stochasticity into gating variables in such a way that the level of fluctuations is dependent on the value of the gating variable. This way we would be able to decrease the fluctuations when the value of the gating variable is approaching 0 or 1 thus decreasing the probability of obtaining values not in the interval [0,1]. This approach is, however, a matter of a future study. Second, none of the freely available neural simulation tools include the possibility to use stochastic differential equations. Presently, self-made simulation software is required which may hinder the use of SDEs in compartmental modeling. Inclusion of a variety of deterministic and stochastic methods in the simulation tools would greatly benefit neuroscientists in simulating the functions of neurons and, ultimately, of neural networks.

In the future, more work will be needed to clarify the roles of different types of noise sources for small, intermediate-size, and large-size neurons, both from experimental and theoretical points of view. As an example, when studying the effects of synaptic input noise the response dynamics of a nerve has been shown to be sensitive to the details of noise model [5]. Moreover, tools from nonlinear dynamics have to be applied to make detailed comparisons between different stochastic methods. Technologies for speeding-up the computations are equally important to develop. The proper addressing of the above-mentioned challenges will enhance our understanding of the role stochasticity has at both microscopic and macroscopic levels.
